# 212. Gonorrhea and Chlamydia Opt-Out Screening of Justice-Involved Females During Intake at the Los Angeles County Jail: The Pivotal Role of Correctional Health Systems

**DOI:** 10.1093/ofid/ofae631.070

**Published:** 2025-01-29

**Authors:** Nazia Qureshi, Sulma J Herrera, Loren G Miller, Stephen Judge, Charles M Cardenas, Sean O Henderson

**Affiliations:** Los Angeles County Department of Correctional Health Services, Los Angeles, CA; Los Angeles County Department of Health Services, Division of Correctional Health Services, Los Angeles, California; Lundquist Institute at Harbor-UCLA Medical Center, Los Angeles, CA; Harbor-UCLA Medical Center, Torrance, California; Los Angeles County Department of Health Services, Division of Correctional Health Services, Los Angeles, California; Los Angeles County Department of Correctional Health Services, Los Angeles, CA

## Abstract

**Background:**

Chlamydia and gonorrhea are two of the most common sexually transmitted infections (STIs) worldwide, presenting major public health challenges and resulting in billions of dollars in direct medical costs in the U.S. Incarcerated females have a particularly elevated risk of these infections, which can result in significant clinical and public/community health-level sequelae if left untreated. On December 13, 2021, the division of Correctional Health Services began offering opt-out urogenital chlamydia and gonorrhea screening to all newly incarcerated females within the Los Angeles County Jail system.

**Figure:**
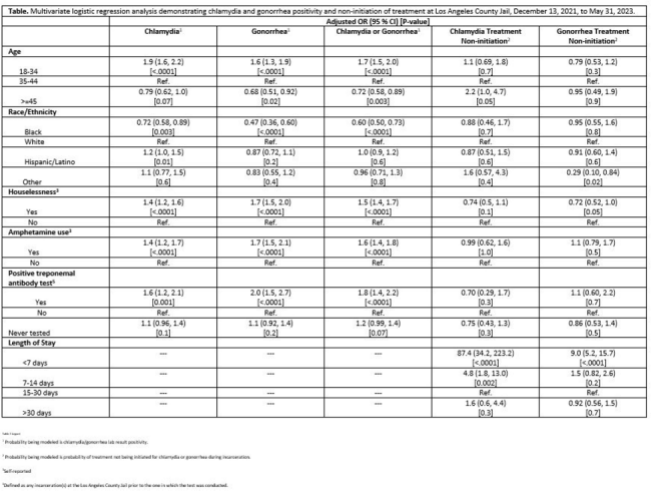

**Methods:**

We retrospectively analyzed electronic health record data for completed urogenital chlamydia/gonorrhea screening among newly incarcerated females between December 13, 2021, and May 31, 2023. We used multivariable logistic regression to examine the association of STIs and treatment non-initiation outcomes with various demographic and self-reported variables.

**Results:**

Of the 13739 women offered STI testing, 10717 (78%) completed screening, with 1151 (11%) having a chlamydial infection, 788 (7%) having a gonococcal infection, and 1626 (15%) having either infection. In a multivariable model, result positivity was associated with age 18-34, reported houselessness, amphetamine use, and history of a positive prior treponemal antibody test. STI treatment non-initiation was associated with shorter length of stay for both chlamydial ([aOR] = 87.4, 95% CI (34.2, 223.2)) and gonococcal ([aOR] = 9.0, 95% CI (5.2, 15.7)) infections.

**Conclusion:**

The STI screening and prevalence among female detainees tested at intake was many-fold higher than that of the general population. The implementation of routine opt-out STI screening and treatment of STI-infected people in carceral settings provides a unique and important opportunity to benefit the health of this high-risk population and has the potential to create a spillover effect that might benefit the surrounding community.

**Disclosures:**

**Loren G. Miller, MD MPH**, Armata: Grant/Research Support|Contrafect: Grant/Research Support|GSK: Grant/Research Support|Merck: Grant/Research Support|Paratek: Grant/Research Support

